# Preparation of stable tau oligomers for cellular and biochemical studies

**DOI:** 10.1016/j.ab.2018.10.013

**Published:** 2019-02-01

**Authors:** Thomas K. Karikari, David A. Nagel, Alastair Grainger, Charlotte Clarke-Bland, Eric J. Hill, Kevin G. Moffat

**Affiliations:** a School of Life Sciences, University of Warwick, Coventry, CV4 7AL, UK; b Midlands Integrative Biosciences Training Partnership, University of Warwick, Coventry, CV4 7AL, UK; c School of Life and Health Sciences, Aston University, Birmingham, B4 7ET, UK

**Keywords:** Tau, Oligomer, Alzheimer's disease, Tauopathies, Maleimide, iPSC-derived neurons

## Abstract

Increasing evidence suggests that small oligomers are the principal neurotoxic species of tau in Alzheimer's disease and other tauopathies. However, mechanisms of tau oligomer-mediated neurodegeneration are poorly understood. The transience of oligomers due to aggregation can compromise the stability of oligomers prepared *in vitro*. Consequently, we sought to develop an efficient method which maintains the stability and globular conformation of preformed oligomers. This study demonstrates that labeling a single-cysteine form of the pro-aggregant tau four-repeat region (K18) with either Alexa Fluor 488-C5-maleimide or *N*-ethylmaleimide in reducing conditions stabilizes oligomers by impeding their further aggregation. Furthermore, the use of this approach to study the propagation of labeled extracellular tau K18 oligomers into human neuroblastoma cells and human stem cell-derived neurons is described. This method is potentially applicable for preparing stabilized oligomers of tau for diagnostic and biomarker tests, as well as for *in vitro* structure-activity relationship assays.

## Highlights

• The transient nature of tau aggregation makes it difficult to maintain the stability of preformed oligomers.
• Efficient labeling of tau K18 with Alexa Fluor-488-C5-maleimide or *N*-ethyl maleimide stabilizes the resulting oligomers.
• Oligomers applied exogenously are propagated intracellularly by cultured human iPSC neurons and neuroblastoma cells.
• Oligomer preparation by maleimide labeling allows mechanistic studies of tau aggregation and its link to neurodegeneration.

## 1 Introduction

The gradual intracellular accumulation of tau aggregates into neurofibrillary tangles (NFTs) is a clinical hallmark for diagnosing and staging Alzheimer's disease (AD) [1]. Nonetheless, tau pathology likely develops several years prior to symptom onset [2]. Indeed, cell death and synaptic lesions have been observed independent of NFT formation [3,4], prompting a need for tau biomarkers that better correlate with disease onset and progression [5]. Increasing evidence suggests that the activities of low molecular weight (LMW) oligomers, formed during the early stages of tau aggregation, most likely associate with neurotoxicity that leads to AD [[6], [7], [8]]. For example, tau oligomers induce mitochondrial, synaptic and memory defects [7] while insoluble filaments have been postulated to provide neuroprotective functions by sequestering toxic oligomers into inert filaments [9]. Many biochemical and toxicity studies of tau use oligomers produced from recombinant sources due to their ability to replicate important aggregative and pathophysiological effects of brain-derived tau oligomers [[10], [11], [12]]. However, the transient formation and subsequent aggregation of LMW tau oligomers present a daunting challenge for their preparation and characterization. Several tau oligomer preparation protocols, including those which have sought to address some of the existing difficulties, have been described [[13], [14], [15], [16]]. Nonetheless, a critical persisting challenge is the stability of the resulting oligomers. For example, oligomeric forms of the central nervous system-specific full length (2N4R) tau isoform prepared by seeding monomer aggregation with preformed alpha synuclein or amyloid beta oligomers lose their stability and aggregate into filaments within 48 h of incubation [15]. This suggests that oligomers prepared *in vitro* are unstable and are prone to further aggregation, which may compromise experimental outcomes and reproducibility due to the distinct conformations that tau protein adopts at different stages of the aggregation process [17,18]. This instability has previously been demonstrated for amyloid beta, another AD-linked protein, which is capable of undergoing detectable aggregation even when stored at an ultra-low temperature (−80 °C) for long periods [19]. This suggests that instability is a major problem of the two most-characterized AD-associated proteinopathic proteins when prepared *in vitro*.

**Fig. 1:**
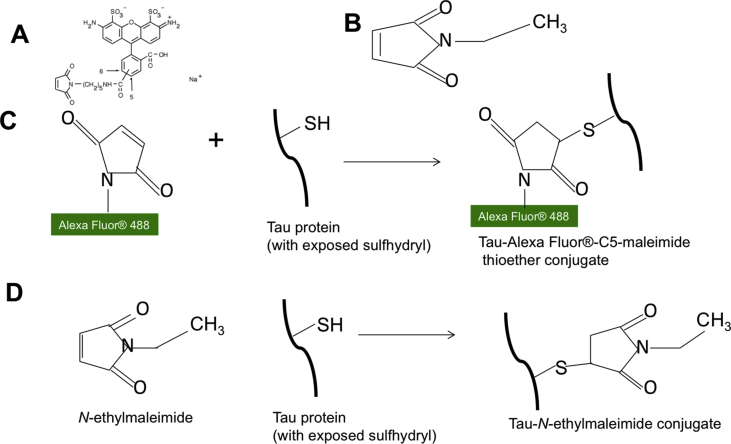
**Schematic illustration of maleimide conjugation to the tau K18 protein.** (A) Molecular structure of AF-maleimide, as provided by the vendor [42]. (B) Molecular structure of NEM. (C) Illustration of the reaction of the maleimide functional group of AF-maleimide with the sulfhydryl group of tau K18 to produce a stable thioether conjugate. (D) Illustration of the specific binding of NEM with the sulfhydryl group of tau K18 to produce a tau K18-NEM conjugate.

Tau aggregation occurs by two known mechanisms, namely: (i) cysteine-dependent polymerization through intermolecular disulfide bonding [20,21], and (ii) cysteine-independent polymerization, occurring through the hexapeptide motifs (^275^VQIINK^280^, ^306^VQIXXK^311^ and ^337^VEVKSE^342^) and non-covalent bonding [[22], [23], [24], [25]]. Although either mechanism would in principle be sufficient, aggregation efficiency is increased in the presence of both [21]. In this study, it was hypothesized that labeling cysteine residues in tau monomers would extend the stability of the oligomers formed by blocking cysteine-dependent aggregation. Since maleimide irreversibly binds cysteine residues, it was postulated that efficient labeling of cysteine residues (Fig. 1) would generate stable tau oligomers. Here, a simple approach of preparing stable LMW tau oligomers by labeling cysteine residues with commercially available derivatives of maleimide, namely Alexa Fluor 488-C5-maleimide (AF-maleimide) and *N*-ethylmaleimide (NEM), is described. Furthermore, the application of the fluorescence property of AF-maleimide-labeling to study the uptake of tau K18 oligomers by SH-SY5Y cells and human induced pluripotent stem cell (hiPSC)-derived neurons is described.

## 2 Materials and methods

### 2.1 Molecular biology and protein purification

The pProEx-HTa-Myc-K18 plasmid used for the recombinant expression of tau K18 was used as previously described [26]. Cysteine modifications (C291A, C322A, I260C) were introduced into the encoded protein to prevent potential functional interference of the maleimide label. Site directed mutagenesis was performed and verified as previously described [26]. 6xHis/c-Myc-tagged K18 was transformed into and expressed in BL21 (DE3)*pRosetta cells and the expressed protein purified using immobilized metal affinity chromatography following a previously-described protocol [26].

### 2.2 Preparation of labeled tau K18 oligomers

Purified tau K18 was treated with 5× molar excess of tris(2-carboxyethyl)phosphine (TCEP) for 1 h, and then with 4× molar excess AF-maleimide (#A10254, Molecular Probes) or NEM (#23030, Thermo Scientific) overnight, in the presence of 10 mM sodium phosphate buffer pH 7.4. The protein was diluted in buffer to a final concentration of 55 μM. Free fluorophore and reducing agent were removed by extensive dialysis against dialysis buffer (50 mM Tris HCl pH 7.5, 100 mM NaCl) in a Slide-A-Lyzer™ MINI Dialysis device (10 K MWCO; Thermo Scientific), with the reservoir perforated and placed in a container large enough to accommodate a 2 L volume of buffer. The buffer was changed every 2 h for 10 h. Labeling was confirmed with non-denaturing sodium dodecyl sulfate polyacrylamide gel electrophoresis (SDS-PAGE) followed by ultraviolet light exposure. Labeling efficiency was measured spectrophotometrically using Beer's law and the molar extinction coefficient of 72,000/cm/M for AF-maleimide. Unlabeled control samples were prepared identically except that equal volume of 10 mM sodium phosphate buffer pH 7.4 was used in place of the maleimide label. The entire labeling process was performed either at 4 °C or at room temperature (RT).

### 2.3 Transmission electron microscopy (TEM)

Formvar/carbon-coated 300-mesh copper grids (#S162, Agar scientific, UK) were glow-discharged using the ELMO system from Cordouan Technologies. Five microliters of labeled or unlabeled K18 preparations were pipetted onto the grid and allowed to bind for 1 min. Excess samples were removed with a strip of filter paper, and 5 μl of 2% uranyl acetate added for 1 min. After removing the excess stain with a strip of filter paper, the grids were imaged using a JEOL-2100F transmission electron microscope.

### 2.4 Dot blotting

Each of the labeled and unlabeled control samples (27.5 μM) was mixed with 125 μM heparin, and incubated at 37 °C in an Eppendorf Mastercycler gradient thermocycler for 48 h. Two microliter aliquots were spotted onto a piece of nitrocellulose membrane, air dried, blocked with 10% non-fat milk in phosphate buffered saline (PBS) containing 0.05% Tween for 30 min at RT, and subsequently washed five times with 10% Tris buffered saline (TBS)-Tween. The membrane was incubated for 2 h with primary antibody (total tau #A0024, Dako or T22, Merck) and washed five times with 10% TBS-Tween. The membrane was then treated with secondary antibody (anti-rabbit immunoglobulin G; 31450, Thermofisher) for 2 h. Antibodies were used at 1:1000 dilutions in the PBS-Tween buffer. Thereafter, the membrane was washed five times as previously, developed with an electrochemiluminescent detection kit (Amersham), exposed to an X-ray film for 2 min and the blots developed in an AGFA Curix 60 processor (Agfa Healthcare, Greenville, SC, USA). Blot intensities were analyzed using ImageJ and the non-parametric data statistically compared with the Mann-Whitney test in GraphPad Prism 7 at the 95% confidence interval.

### 2.5 SH-SY5Y cell culture

SH-SY5Y cells were purchased from Sigma Aldrich UK (#94030304) and maintained on 1:1 minimal essential medium (MEM)/F12 Ham medium containing 1% l-Glutamine, 15% foetal bovine serum, and 1% antibiotic antimycotic acid (10,000 units penicillin, 10 mg streptomycin and 25 μg amphotericin B). All reagents were obtained from Sigma Aldrich. Cells between passages two and ten were used for all experiments. The cells were seeded at 200,000 cells/ml in CellView™ Advanced Tissue Culture dishes (#627975, Greiner Bio-One) in the presence of AF-maleimide-labeled tau K18 oligomers dissolved in the culture medium to 5 μM. Following 24 h incubation at 37 °C, 5% CO_2_, the spent medium was removed, the cells washed with warm PBS and fresh tau-free medium containing 2 μM Hoechst 33342 (#H21492, Molecular Probes) and CellMask Deep Red (1:1000 dilution, #C10046, Thermofisher). Confocal microscopy imaging of internalized tau was performed after 30 min incubation at 37 °C, 5% CO_2_ using an LSM 710 microscope (Leica).

### 2.6 hiPSC-derived cortical neurons

Neural precursor cells (#ax0016) from cord blood CD34 ^+^ cells of a healthy, newborn female donor were purchased from Axol Bioscience, Cambridge, UK. Tissue culture grade 12-well plates (Corning, New York, USA) were pre-coated with 250 μl/cm^2^ ReadySet reagent (Axol Bioscience), incubated at 37 °C, 5% CO_2_ for 45 min and rinsed four times with double distilled water. Following this, Surebond reagent (Axol Bioscience) was diluted in Dulbecco's PBS (DPBS), added to the plates at 200 μl/cm^2^ and incubated at 37 °C, 5% CO_2_ to equilibrate for 1 h. Next, stem cells were seeded at a low density (25,000 cells/cm^2^) on plasma-cleaned 13 mm glass coverslips, incubated at 37 °C, 5% CO_2_ and media changed every other day with Axol Neural Maintenance Medium kit (#ax0031, Axol Bioscience) for 14–16 days prior to using the cells for experiments. Neuronal differentiation was monitored using phase contrast images obtained periodically with an EVOS XL Core Imaging System (Life Technologies) and the expression of neuron-specific markers monitored by immunohistochemistry (data not shown). Tau oligomers were diluted to 5 μM in Axol Neural Maintenance medium, supplied to neurons and then incubated for 24 h at 37 °C, 5% CO_2._ After removing the spent medium, the neurons were washed with DPBS, and new medium added.

Neurons were then fixed for 30 min with 4% paraformaldehyde, washed two times with DPBS, and rinsed with permeabilizing solution (DPBS containing 0.2% Triton). Afterwards, the fixed neurons were incubated for 1 h in blocking buffer (DPBS with 0.2% Triton and 2% BSA). This was followed by another 1 h incubation in the blocking buffer containing primary antibody and Hoechst. Excess antibody was removed by washing thrice with blocking buffer only for 5 min each time. Subsequently, the neurons were incubated in blocking buffer containing secondary antibody for 1 h, and the blocking buffer wash steps repeated. Next, the slides were rinsed with distilled water and dropped into ProLong Gold Antifade mounting medium (#P36934, ThermoFisher Scientific). Neurons were imaged with a Leica STP 6000 confocal microscope after at least 24 h of curing.

## 3 Results and discussion

This study used the K18 fragment (tau microtubule (MT) binding region; residues 244–372), since a demonstration of oligomer stability for this aggregation-prone functional tau region would in principle make the method applicable to the full-length isoforms which are less aggregation-prone [27] and may therefore be easier to stabilize. Due to their close proximity to residues that directly bind MTs [28], the two native cysteine residues in tau K18 were modified to alanine and a new one introduced at the N-terminus of the MT-binding region (see the *Materials and Methods* section). This approach was to ensure efficient labeling of a single cysteine residue without potential interference of MT binding [17,29,30].

**Fig. 2:**
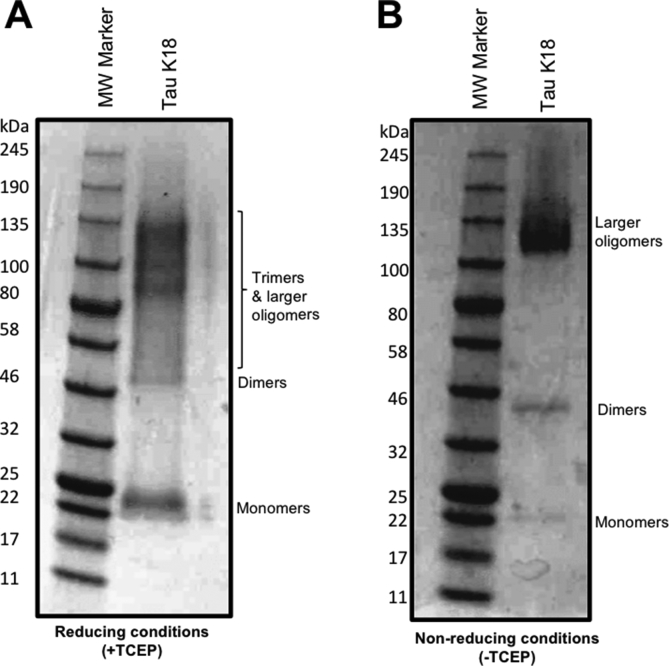
**Representative non-denaturing SDS-PAGE images demonstrating the outcome of AF-maleimide labeling of tau K18 in reducing and non-reducing conditions.** Migration of tau K18 labeled with AF-maleimide with (A) or without (B) prior TCEP treatment. The figures show representative (n = 3) non-denaturing SDS-PAGE analysis of tau aggregate patterns in the two labeling methods.

**Fig. 3:**
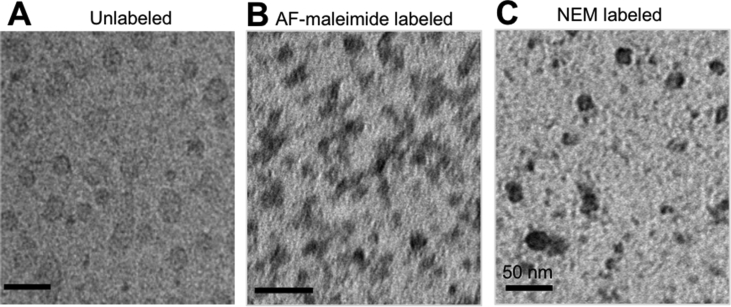
**Structural characterization of tau K18 oligomers stabilized by labeling with maleimide derivatives.** Representative TEM micrographs of globular tau K18 oligomers prepared: (A) without labeling, (B) with AF-maleimide labeling, or (C) with NEM labeling. Scale bars = 50 nm for all images.

The K18 tau construct was cloned into pProEx, transformed into BL21 (DE3) *Escherichia coli*, expressed, purified and biochemically characterized as described [26]. Oligomers were prepared by initially treating freshly-purified proteins with the reducing agent TCEP for 1 h to ensure all proteins were monomeric. Thereafter, the exposed sulfhydryl groups were labeled with AF-maleimide and the reaction allowed to proceed at 4 °C overnight followed by extensive dialysis to remove excess dye and TCEP. This “monomerization-labeling-oligomerization” strategy ensured accessibility of the labeling site to the maleimide dye. The overnight incubation in cold conditions allowed for limited aggregation of labeled monomers into LMW oligomers. Control samples labeled without TCEP treatment lacked the uniform oligomer distribution observed in those TCEP-treated before labeling (Fig. 2). The labeled proteins aggregated into globular, LMW oligomers (Fig. 3), akin to what is found in the brains of AD patients [6] and those reported from other recombinant tau oligomerization protocols [15]. No discernible difference in oligomerization patterns was observed between the labeled and unlabeled proteins (Fig. 3) as all samples were capable of forming oligomers. However, oligomers assembled from unlabeled tau K18 were unstable as explained below.

**Fig. 4:**
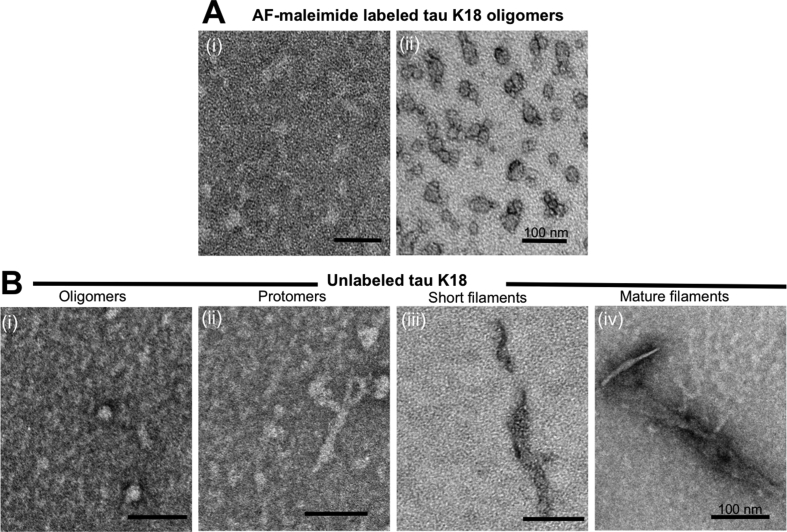
**Labeling with AF-maleimide stabilizes tau K18 oligomers.** (A) Representative electron micrographs of AF-maleimide-labeled tau K18 structures identified after co-incubation with heparin at 37 °C for 48 h. Figures A(i) and A(ii) show globular oligomers with different degrees of negative staining. (B) Representative TEM micrographs of aggregates identified for the unlabeled control samples treated similarly to the test samples. Globular oligomers (i), protomers (ii), short and mature fibrils (iii and iv respectively) were observed, suggesting heparin-induced structural transition to form fibrils. n = 2, with images taken from at least 5 different areas of each TEM grid. Scale bars = 100 nm for all images.

To test the hypothesis that cysteine labeling interferes with tau aggregation, the AF-maleimide labeled oligomers and unlabeled controls were challenged with the aggregation inducer heparin and heated at 37 °C without shaking for 48 h. After incubation, aliquots were taken from each sample following quick mixing and analyzed by negative-stain TEM. The labeled proteins existed mostly as globular oligomers (Fig. 4A), appeared mainly trimeric/tetrameric in distribution (Fig. 2A), and were of similar structure as those imaged prior to heparin and heat treatment (Fig. 3B). By contrast, the unlabeled protein underwent further aggregation to form mature insoluble filaments, via intermediate protomers and early-stage filaments (Fig. 4B). These results indicate that AF-maleimide labeling stabilizes tau oligomers and prevents their aggregation and conformational change into paired helical filaments.

**Fig. S1:**
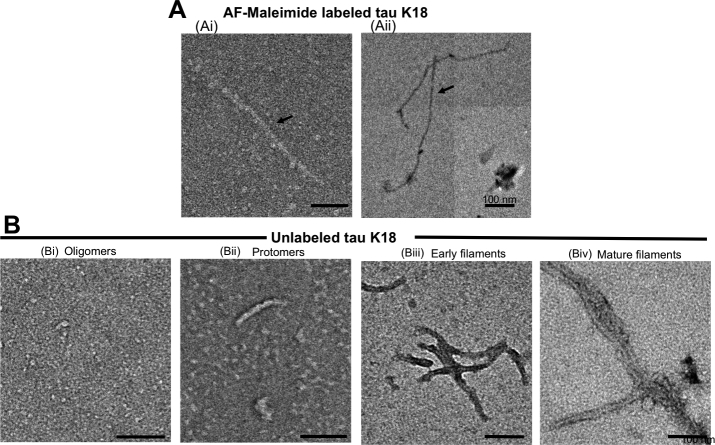
**AF-maleimide labeling of tau K18 at RT generates a mixture of globular oligomers and fibrillar aggregations.** (A) Representative TEM micrographs of tau K18 labeled with AF-maleimide at RT showing presence of both globular oligomers and fibrils (shown by arrows). (B) Unlabeled control tau K18 samples aggregated into a range of pre-fibrillar and fibrillar structures. Scale bars = 100 nm.

Performing the reaction under cold conditions appeared critical to the success of the new method. Contrary to the dominance of globular oligomers when labeled at 4 °C (Fig. 3, Fig. 4), a mixture of globular oligomers and fibrils was observed when the reaction was performed at RT (Fig. S1). This phenomenon may be explained by the higher labeling efficiency at 4 °C compared to at RT (97% and 82.5% respectively); there is a much higher proportion of unlabeled starting material at RT capable of aggregating into fibrils. This data is in agreement with previous reports showing high AF-maleimide labeling efficiencies at 4 °C [31,32], and also explains why fibrils can be detected among tau proteins labeled at RT or 37 °C [29,30].

**Fig. 5:**
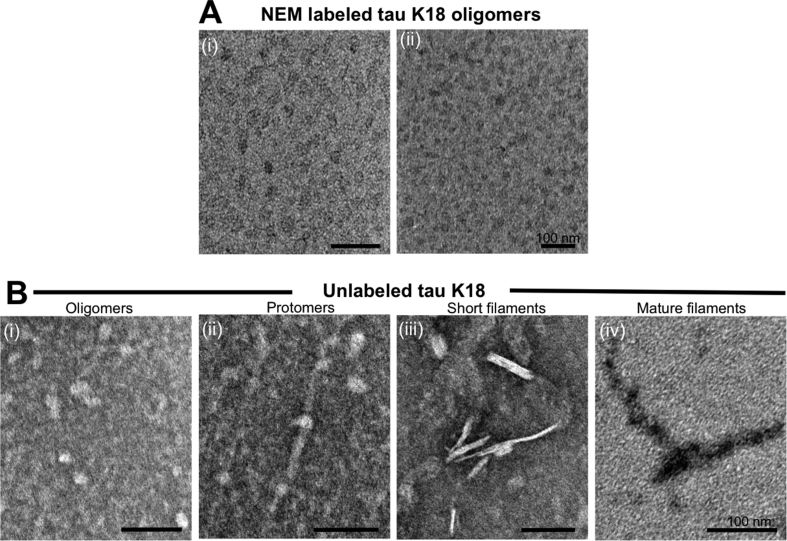
**NEM labeling stabilizes tau K18 oligomers in their globular conformation.** NEM-labeled and unlabeled control tau K18 samples were each incubated with heparin at 37 °C for 48 h, and aliquots analyzed using negative-stain TEM. (A) Representative TEM micrographs of NEM-labeled tau K18 aggregates. The globular nature of these non-fibrillar aggregates in A(i) and A(ii) suggests oligomer stabilization. (B) Representative TEM micrographs of endpoint samples demonstrating the aggregation of unlabeled tau K18 into oligomers, protomers, short and mature fibrils. n = 2, with images taken from at least 5 different areas of each grid. Scale bars = 100 nm for all images.

Next, we asked if the observed oligomer-stabilizing property of AF-maleimide was shared by other maleimide derivatives. For this reason, the alkylating agent NEM which reacts in a similar mechanism as AF-maleimide with sulfhydryls to form stable thioesters was tested. NEM labeling of tau K18 was performed as described for AF-maleimide. To test their stability, the formed oligomers were treated with heparin, incubated at 37 °C for 48 h and thereafter characterized by TEM. It was observed that, like AF-maleimide, NEM labeling stabilized tau oligomers also appeared to block further aggregation into filaments (Fig. 5A and B).

**Fig. 6:**
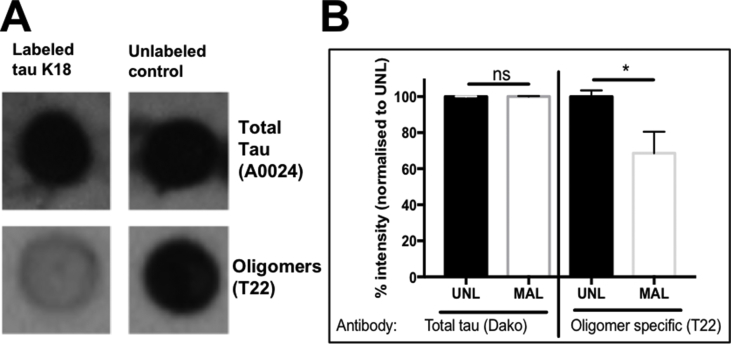
**Immunological analysis of the conformation of AF-maleimide-labeled tau K18 oligomers using dot blotting.** (A) Representative dot blot data for the reactivity of labeled and unlabeled tau K18 to two tau antibodies (A0024, top panel) and T22 (bottom panel). (B) Semi-quantitative analysis of dot blot intensity using Image J. UNL = unlabeled; MAL = AF-maleimide labeled. Mann Whitney test, * = p < 0.05, ns = not significant (n = 4). Data expressed as mean ± standard deviation.

**Fig. 7:**
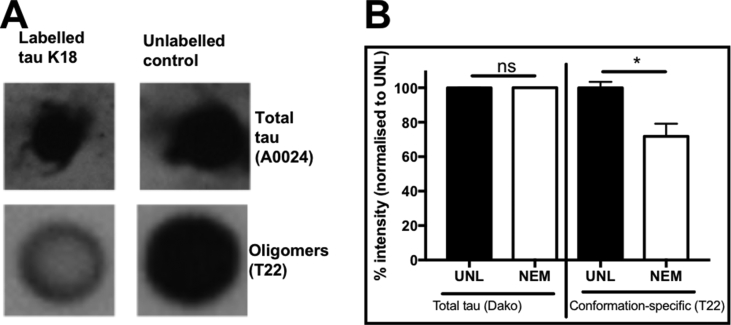
**Dot blot analysis of the conformation of NEM-labeled tau K18 oligomers.** (A) Representative dot blot data for the immuno-reactivity of labeled and unlabeled tau K18 to two tau antibodies (A0024, top panel) and T22 (bottom panel). (B) Semi-quantitative analysis of dot blot intensity using Image J. UNL = unlabeled; NEM = NEM labeled. Mann Whitney test, * = p < 0.05, ns = not significant (n = 4). Data expressed as mean ± standard deviation.

Subsequently, the effect of maleimide labeling on the conformation of tau K18 oligomers was investigated. The immunoreactivity of oligomer preparations labeled with AF-maleimide or NEM was probed with an oligomer conformation-specific antibody (T22) and a total tau antibody (A0024, Dako) as a control in dot blotting experiments. Oligomers labeled using either method gave near-identical total tau reactivity to the unlabeled control (Fig. 6, Fig. 7). However, the labeled proteins were consistently less reactive to the T22 antibody (Mann Whitney test, p < 0.05 in both cases; Fig. 6, Fig. 7). This suggests that the conformation of the unlabeled tau K18 oligomers recognized by the T22 antibody was altered by the maleimide labeling. A few reasons could have accounted for this reduced recognition. Firstly, the maleimide labels may have masked the antibody epitope thereby reducing accessibility and binding of the antibody. A similar outcome has recently been reported from a pharmacological inhibition of tau oligomer aggregation where binding of the phenothiazine compound Azure C also reduced T22 reactivity [33]. Secondly, the antibody preferably recognises larger oligomers over LMW oligomers, such as those found in the labeled samples. As the unlabeled samples aggregated further into fibrils, it can be postulated that they may have consisted of larger pools of higher molecular weight oligomers which would be responsible for their stronger reactivity. T22 is thought to selectively recognize non-fibrillar tau oligomers [8] but it may not be able to discriminate between oligomers of different sizes which share the same conformational epitope. The stronger immuno-reactivity recorded for the unlabeled samples may be due to differences in non-fibrillar aggregated proteins compared to the labeled (Fig. 6, Fig. 7). Thirdly, since the T22 antibody can also cross-react with fibrils [8], the presence of fibrils in the unlabeled samples may have increased the reactivity of the T22 antibody in the unlabeled samples. As the only difference between the labeled and the unlabeled tau K18 samples was the extent of aggregation as evidenced by TEM analysis, it can be concluded that AF-maleimide labeling reduces reactivity of tau K18 oligomers to the T22 conformation-dependent antibody, which may be due to their restricted aggregation. This finding along with those of others [32] suggests care should be taken when assessing oligomerization using the T22 antibody alone.

**Fig. 8:**
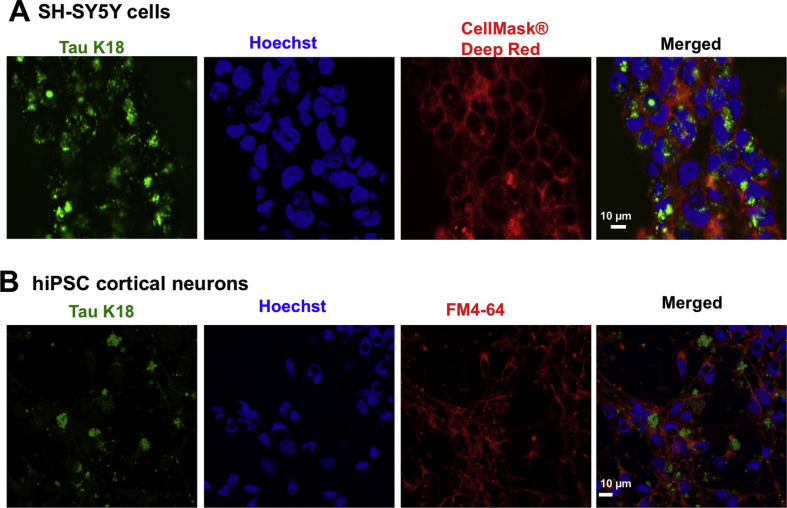
**Extracellularly-applied tau K18 oligomers are internalized by SH-SY5Y neuroblastoma cells and hiPSC-derived neurons.** (A) Internalization of AF-maleimide-labeled tau K18 oligomers in SH-SY5Y neuroblastoma cells. (B) Internalization of extracellular oligomers was also studied in hiPSC-derived neurons, revealing their localization both in neurites and the cell soma. Scale bars = 10 μm for all images.

A mechanism proposed for the propagation of AD pathology is the transmission of toxic tau proteins from one neuron to the other in specific brain regions [[34], [35], [36]]. A critical step in this inter-neuronal transmission model is the uptake of extracellular tau into neighboring neurons, followed by their interaction with endogenous proteins and subsequent release of the resulting aggregates to initiate new internalization cycles [[34], [35], [36]]. For better understanding of the molecular cascade of events occurring in this process, real-time microscopic visualization of live cells treated with tau is necessary. As oligomers are thought to be a crucial species regulating the inter-neuronal transmission of tau pathology [6,8,10,11], the molecular fluorescence property of AF-maleimide was used to study the internalization of stabilized extracellular tau K18 oligomers into cultured SH-SY5Y human neuroblastoma cells. AF-maleimide oligomers were prepared as described above, diluted in extracellular medium to 5 μM final concentration, supplied exogenously to cultured cells and incubated for 24 h. Subsequently, the spent medium was removed, the cells washed with warm phosphate buffered saline to remove non-internalized tau and new tau-free medium containing nuclear and cell membrane markers added. The internalized tau was then visualized using confocal microscopy. Remarkably, internalization of fluorescent extracellular tau was observed, with cells containing these internalized proteins exhibiting diverse morphological phenotypes: in some cells diffused cytoplasmic distribution of labeled protein was observed, with more localized punctate staining in other cells (Fig. 8A). These phenotypes were similar to those reported for labeled tau K18 internalized in the same neuroblastoma cell line [30], as well as other human cell lines [37]. To extend the use of these fluorescently labeled oligomers toward a more physiologically-relevant neuronal model, hiPSC-derived cortical neurons were prepared and seeded with 5 μM extracellular tau oligomers. Following 24 incubation, the neurons were fixed using paraformaldehyde. Maximum projection of z-stack confocal microscopy images revealed that tau oligomer internalization likely occurs by endocytosis (as oligomers were encapsulated by the vesicular marker FM4-64^®^) and that the inclusions accumulated both in the cell soma and neurites, hence providing insights into the subcellular localization of tau oligomer inclusions in addition to their morphological phenotypes (Fig. 8B). Together, these findings demonstrate that maleimide stabilization offers a unique strategy for the characterization of cellular internalization of tau oligomers and to screen for therapeutic agents against this disease-relevant process.

**Fig. 9:**
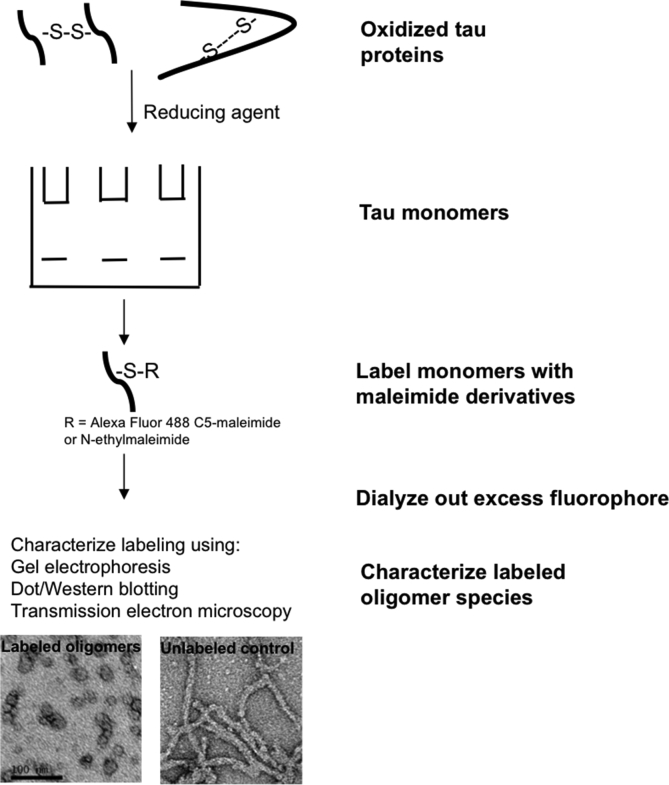
**Schematic illustration of the facile method of tau oligomer stabilization described in this study.** The method involves first treating highly-purified monomers with the reducing agent TCEP to monomerize apparent oligomers. Monomers are then labeled overnight with either maleimide derivative (AF-maleimide or NEM), followed by removal of excess fluorophore by dialysis. Labeled oligomers can then be characterized using an array of biochemical and biophysical tools, including non-denaturing SDS-PAGE, dot/Western blotting and TEM. Oligomer stabilization can be studied by inducing aggregation with heparin and studying the structures and conformation of the aggregates formed by TEM and dot blotting respectively, or by similar techniques.

Here, we have demonstrated that efficient labeling of tau K18 with AF-maleimide or NEM provides the advantage of stabilizing the resulting oligomers. Interestingly, a recent study by Chen et al. (2018) has corroborated our findings, showing that NEM labeling of the single cysteine residue at amino acid position 322 of the shortest tau isoform in the adult human brain (0N3R) prevents its H_2_O_2_-induced aggregation [38]. Our proposed strategy (Fig. 9) ensures that preformed tau oligomers remain in their desired authentic oligomeric state for extended periods of time, offering an exceptional opportunity to advance downstream investigations into the biochemical, biophysical and neurobiological aspects of the association between tau aggregation and neurodegeneration. Moreover, the method is simple, time-efficient and does not require expensive pre- and post-treatment stages. The proposed approach is potentially applicable to brain-derived tau oligomers, and could support studies of mechanistic and conformational changes in tau oligomers that may alter the protein's neurotoxicity and drug-reactivity profiles. Additionally, the method can likely be extended to other proteinopathic proteins containing cysteine residues, such as huntingtin and cysteine-containing amyloid beta [39]. We have applied this approach to obtain novel insights into the subcellular internalization and neuronal transmission of exogenous tau K18. This has allowed an analysis of the effects of tau mutant variants implicated in diseases such as frontotemporal dementia on the protein's neuronal internalization. Our results indicate that this method provides a quick and reliable approach of monitoring cellular internalization of exogenous proteins in real time, and therefore offers an advantage over other fluorescent tags especially for small proteins like tau K18. Furthermore, the neuronal uptake and secretion of extracellular tau oligomers are thought to be regulated by protein-cell membrane interactions, details of which are still unclear [40,41]. This oligomer stabilization strategy offers a straightforward approach to study conformational and aggregation dynamics of protein-membrane interactions both at the single and bulk molecular levels using fluorescent spectroscopy and cell biology techniques. Additionally, stabilized oligomers are good immunogen candidates for antibody production against tau oligomer-targeted immunotherapy.

## Competing interests

The authors declare no competing interests.

## Author contributions

TKK, DAN, EJH and KGM designed the research. TKK, DAN, AG, and CC-B conducted the experiments and analyzed the data. TKK, DAN, EJH and KGM wrote the paper.

## Abbreviations

AD: Alzheimer's disease
AF-maleimide: Alexa Fluor 488-C5-maleimide
DPBS: Dulbecco's phosphate buffered saline
LMW: low molecular weight
MT: microtubule
NEM: N-ethylmaleimide
NFT: neurofibrillary tangle
PBS: phosphate buffered saline
RT: room temperature
SDS-PAGE: sodium dodecyl sulfate – polyacrylamide gel electrophoresis
TCEP: tris(2-carboxyethyl)phosphine
TBS: Tris buffered saline
TEM: transmission electron microscopy
